# Assessing Placental Shear Wave Elastography as a Tool for Evaluating Preeclampsia

**DOI:** 10.7759/cureus.68553

**Published:** 2024-09-03

**Authors:** Rajesh Kuber, Eshan Chetan Durgi, Neeha A Jhala, Urvashi R Jainani, Rahul Mane, Rohan N Shah, Amanya Shukla

**Affiliations:** 1 Radiology, Dr. D. Y. Patil Medical College, Hospital & Research Centre, Dr. D. Y. Patil Vidyapeeth, Pune, IND; 2 Radiodiagnosis, Dr. D. Y. Patil Medical College, Hospital & Research Centre, Dr. D. Y. Patil Vidyapeeth, Pune, IND; 3 Obstetrics and Gynecology, Dr. D. Y. Patil Medical College, Hospital & Research Centre, Dr. D. Y. Patil Vidyapeeth, PUNE, IND; 4 Radiodiagnosis, Aaxis Diagnostic Centre, Sangola, IND

**Keywords:** fetal growth restriction, prediction of preeclampsia, hypertensive disorders of pregnancy, preeclampsia, shear wave elastography

## Abstract

Background

Hypertensive disorders of pregnancy, especially its dreaded complication preeclampsia, remain a major cause of morbidity and mortality for both the mother and the fetus. Existing tools for the prediction of preeclampsia remain inadequate in their sensitivity and specificity. Hence, there is an urgent need for a reliable, economically feasible, and objective marker for its diagnosis/early prediction. In this regard, shear wave elastography has shown great promise. Shear wave elastography is a novel method to quantify tissue stiffness, which is objective and has significantly lower inter-observer variability.

Objectives

We aim to quantify the tissue elasticity using point shear wave elastography (pSWE) in the placentas of diagnosed cases of preeclampsia and to compare them with the placentas of healthy controls in order to evaluate if there is a significant statistical difference between the two.

Materials and methods

This comparative study was conducted at the Department of Radiodiagnosis, Dr. D. Y. Patil Medical College, Hospital and Research Centre, Pune, India, from August 2022 to July 2024.

The study included 60 participants, divided into two groups: 30 patients with preeclampsia and 30 healthy pregnancies. Placental stiffness was measured using a Samsung HS70A ultrasound machine (Samsung Electronics Pvt. Ltd., Seoul, South Korea), and pSWE was performed with a curvilinear probe. Data was analyzed using IBM SPSS Statistics for Windows, Version 21 (Released 2012; IBM Corp., Armonk, New York, United States), and the significance of differences between the two groups was assessed using an independent t-test with a p-value of <0.05, considered statistically significant.

Results

The mean placental stiffness, measured in kilopascals (kPa), was significantly higher in the preeclampsia group (11.71 ± 1.52 kPa) in comparison to the healthy group (3.36 ± 0.66 kPa) (p = 0.001). Patients suffering from preeclampsia were found to have significantly higher levels of placental stiffness.

Conclusion

Early diagnosis remains key to managing preeclampsia so that adequate monitoring and treatment could be provided to the patients. Our study showed that there is a significant statistical difference in the placental stiffness in patients with preeclampsia in comparison to a healthy placenta. Hence, shear wave elastography can be used as a supplementary tool to aid in the diagnosis/prediction of preeclampsia.

## Introduction

Hypertensive disorders of pregnancy are among the most significant and intriguing unsolved problems in obstetrics. Hypertensive disorders during pregnancy, alongside hemorrhage and infections, significantly increase maternal mortality and morbidity. They include preeclampsia and eclampsia, chronic hypertension, preeclampsia superimposed on chronic hypertension, and gestational hypertension (definitive evidence of preeclampsia does not develop, and hypertension usually resolves by 12 weeks postpartum). They complicate 2-8% of all pregnancies, leading to more than 50,000 maternal deaths and over 500,000 fetal deaths worldwide [1]. Among hypertensive conditions during pregnancy, preeclampsia syndrome, whether alone or occurring alongside chronic hypertension, poses the highest risk [2]. Preeclampsia refers to new-onset hypertension and proteinuria, or the new onset of hypertension along with significant end-organ damage with or without proteinuria, which typically presents after 20 weeks of gestation or postpartum.

Preeclampsia can virtually involve every organ system. Due to its severity, prediction of preeclampsia has become a major avenue of research to help in mitigation of its effect and possible prevention. Several biological, biochemical, and biophysical markers have been suggested for predicting its onset; however, testing strategies have shown low sensitivity and poor positive predictive value for preeclampsia [2]. In sonography, uterine artery or fetal transcranial velocimetry is most commonly used to predict preeclampsia. However, currently, no screening tests are predictably reliable, valid, and economical [3].

Elastography is an imaging technique used to assess the elasticity of tissue through quantitative or semiquantitative measurements [4]. Shear wave elastography is a novel method of non-invasive ultrasound to quantify tissue elasticity (tissue stiffness) while evaluating tissue pathology. This method has already proven its usefulness in organs like the liver. The tissue stiffness is measured by Young’s modulus and is expressed in pascals (Pa) or kilopascals (kPa).

Placental stiffness is thought to vary between healthy pregnancies and pregnancies complicated by preeclampsia [5]. Hence, shear wave elastography may be of use in quantifying the elasticity of the placenta and thereby helping to diagnose/predict preeclampsia disease.

Our study aims to quantify the tissue elasticity in the placentas of diagnosed cases of preeclampsia and to compare them with the placentas of healthy controls in order to evaluate if there is a significant statistical difference between the two, which can further be used in predicting preeclampsia disease at an earlier gestational age so as to start appropriate management in a timely fashion to reduce morbidity and mortality associated with preeclampsia disease.

## Materials and methods

This comparative study was conducted at the Department of Radiodiagnosis, Dr. D. Y. Patil Medical College, Hospital and Research Centre, Pune, India, from August 2022 to July 2024. This study included 60 participants. The Institutional Ethics Subcommittee of Dr. D. Y. Patil Medical College, Hospital and Research Centre, approved this study (Reference number: IESC/PGS/2022/165).

Inclusion and exclusion criteria

Inclusion criteria used were pregnant women diagnosed with preeclampsia (as per the American College of Obstetricians and Gynecologists) between 24 and 40 weeks gestation, and healthy pregnant women as controls. Exclusion criteria included placental anomalies; insufficient placental adherence; hematomas and gross calcifications of the placenta; multiple gestations; fetal chromosomal or major structural anomalies; severe anemia; posterior placental location in the uterus to avoid inadequate elasticity calculations; and pregnancies complicated by other major disorders such as gestational diabetes mellitus and autoimmune diseases.

Study procedure

The Department of Obstetrics and Gynecology at Dr. D.Y. Patil Medical College, Hospital and Research Institute, Pune, referred 30 diagnosed cases of preeclampsia. We randomly selected 30 healthy pregnant women as controls from the pool of OPD patients visiting the department of radiodiagnosis for routine obstetric scans. Consent was obtained from all the women participating in the study. Appropriate forms (Form F) as per the PC-PNDT Act, 2003 were filled out and duly submitted to appropriate authorities. The radiologist performing the scan was blinded to the diagnosis of preeclampsia and other studies (obstetric Doppler, laboratory results).

Technique

A Samsung HS70A ultrasound machine (Samsung Electronics Pvt. Ltd., Seoul, South Korea) was used, using a curvilinear transducer equipped with shear wave elastography.

Demographic details were collected: age (in years), parity, gestational age (in weeks), and patient registration number (PRN) assigned by the institute were also noted so that the name of the patient, name of spouse/parent, city/district of residence, and contact number can be traced from the hospital information system (HIS) whenever needed. Clinical details were noted so as to make sure that the exclusion criteria were followed.

Shear wave elastography: Initial B-mode imaging was performed to look for placental position and to rule out placental anomalies and abnormal placental adherence. An area of interest was then located using B-mode imaging. Shear wave elastography was then performed with the patient in the supine position. A square-shaped region of interest (ROI) of size 1 cm x 1 cm, was placed in the elastography window. Care was taken to place the ROI at least 1 cm away from vessels and cystic areas within the placenta. Tissue elasticity was measured in kilopascals (Kpa) while the patient suspends respiration. The mean elasticity of 7-10 samples from fetal and maternal surfaces in the peripheral and central regions of the placenta was calculated. For each value, a reliability measurement index (RMI) of >0.5 was used, which has a strong correlation with reproducible measurements. Interquartile range (IQR) was kept below 30%. Mean tissue stiffness (in Kpa) was then noted.

**Figure 1:**
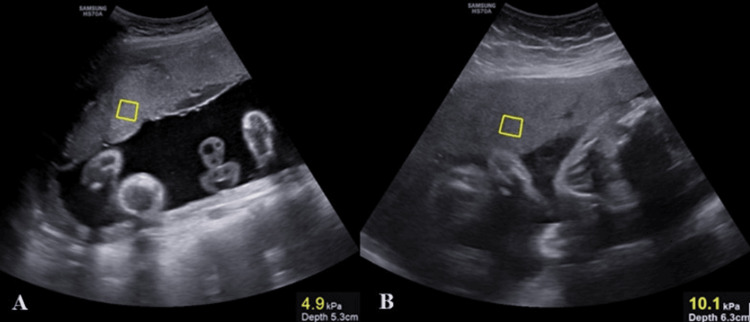
pSWE image showing placental tissue stiffness in the region of interest (ROI) in two patients. A: showing placental stiffness measurement in a 24-year-old G2P1L1 (29 weeks’ gestation); B: showing placental stiffness in a 24-year-old primigravida (28 weeks’ gestation), diagnosed case of preeclampsia pSWE: point shear wave elastography

Figure 1 displays a pSWE image depicting the measurement of placental stiffness in kPa in two patients.

Statistical analysis

Data was entered into Microsoft Excel (Microsoft Corporation, Redmond, Washington, United States). The analysis was conducted using IBM SPSS Statistics for Windows, Version 21 (Released 2012; IBM Corp., Armonk, New York, United States). Descriptive statistics of the explanatory and outcome variables were calculated by mean, standard deviation for quantitative variables, frequency, and proportions for qualitative variables. Inferential statistics like the chi-square test were applied for qualitative variables to find the association. An independent sample t-test was applied to compare the quantitative parameters between the groups. The level of significance is set at 5%.

## Results

Study population

The study consisted of 60 participants, divided into two groups. Group A: 30 healthy pregnancies (controls); and Group B: 30 patients diagnosed with preeclampsia (cases). Both cases and controls were equally represented in the research population (50%).

Table 1 compares the mean ages of two groups using an independent samples t-test. Group A (controls) consisted of 30 participants with ages ranging from 21.0 to 35.0 years and a mean age of 26.13 years (standard deviation = 3.40). Group B (cases) also consisted of 30 participants, with ages spanning from 21.0 to 38.0 years and a mean age of 27.03 years (standard deviation = 3.71). The mean difference in age between the two groups was -0.90 years, indicating that Group A was, on average, younger than Group B. However, this difference was not statistically significant, with a p-value of 0.331.

**Table 1:** Comparison of the mean age between the groups using independent sample t-test. N: number; all ages mentioned are in years; S.D.: standard deviation

Groups	N	Minimum age	Maximum age	Mean age	S.D	Mean difference	p-value
Group A (controls)	30	21.0	35.0	26.13	3.40	-0.90	0.331
Group B (cases)	30	21.0	38.0	27.03	3.71		

Table 2 shows the distribution of the subjects based on age groups. Among the participants, 46.7% of Group A (controls) and 36.7% of Group B (cases) were aged between 21 and 25 years, contributing to 41.7% of the total sample. In the age group of 26 to 30 years, 46.7% of Group A and 43.3% of Group B were included, making up 45.0% of the total participants. For those aged over 30 years, only 6.7% of Group A fell into this category, compared to 20.0% of Group B, accounting for 13.3% of the entire sample. The chi-square value for the age group distribution was 2.39, with a p-value of 0.302, indicating no statistically significant association in the age distribution between the two groups.

**Table 2:** Distribution of the subjects based on age groups.

Age Groups		Groups		Total
		Group A (controls)	Group B (cases)	
21 to 25 yrs	Count	14	11	25
	%	46.7%	36.7%	41.7%
26 to 30 yrs	Count	14	13	27
	%	46.7%	43.3%	45.0%
> 30 yrs	Count	2	6	8
	%	6.7%	20.0%	13.3%
Total	Count	30	30	60
	%	100.0%	100.0%	100.0%
Chi-square value-2.39				
p-value-0.302				

Parity and gestational age

Table 3 presents the distribution of subjects based on parity for Group A and Group B. In Group A, 56.7% (17 participants) were multiparous, while 43.3% (13 participants) were primiparous. In Group B, 66.7% (20 participants) were multiparous and 33.3% (10 participants) were primiparous. Overall, across both groups, 61.7% of the subjects were multiparous and 38.3% were primiparous. The chi-square value for the parity distribution was 0.635, with a p-value of 0.426, indicating no statistically significant association in the parity distribution between the two groups.

**Table 3:** Distribution of the subjects based on parity.

Parity		Groups		Total
		Group A	Group B	
Multi	Count	17	20	37
	%	56.7%	66.7%	61.7%
Primi	Count	13	10	23
	%	43.3%	33.3%	38.3%
Total	Count	30	30	60
	%	100.0%	100.0%	100.0%
Chi-square value-0.635				
p-value-0.426				

Table 4 compares the mean gestational age in weeks between Group A and Group B using an independent samples t-test. Group A, consisting of 30 participants, had gestational ages ranging from 24.0 to 37.0 weeks, with a mean of 30.23 weeks and a standard deviation of 3.76. Group B, also with 30 participants, had gestational ages ranging from 26.0 to 37.0 weeks, with a mean of 31.97 weeks and a standard deviation of 3.45. The mean difference in gestational age between the two groups was -1.73 weeks, indicating that Group A had a lower average gestational age compared to Group B. However, this difference was not statistically significant, with a p-value of 0.068.

**Table 4:** Comparison of the mean gestational age in weeks between the groups using independent sample t-test. N: number; S.D.: standard deviation

Groups	N	Minimum gestational age	Maximum gestational age	Mean gestational age	S.D	Mean difference	p-value
Group A	30	24.0	37.0	30.23	3.76	-1.73	0.068
Group B	30	26.0	37.0	31.97	3.45		

Placental stiffness

Table 5 compares the mean placental stiffness (kPa) between Group A and Group B using an independent sample t-test. Group A, consisting of 30 participants, had placental stiffness values ranging from 2.4 to 4.6 kPa with a mean stiffness of 3.36 kPa and a standard deviation of 0.66 kPa. In contrast, Group B had placental stiffness ranging from 8.9 to 14.8 kPa, with a mean stiffness of 11.71 kPa and a standard deviation of 1.52 kPa. The mean difference in placental stiffness between the two groups was -8.34 kPa, indicating that Group A had significantly lower placental stiffness compared to Group B. This difference was statistically significant, with a p-value of 0.001.

**Table 5:** Comparison of the mean placental stiffness in kPa between the groups using independent sample t-test. N: number; * : significant

Groups	N	Minimum (kPa)	Maximum (kPa)	Mean (kPa)	S.D.	Mean difference	p-value
Group A	30	2.4	4.6	3.36	0.66	-8.34	0.001*
Group B	30	8.9	14.8	11.71	1.52		

**Figure 2:**
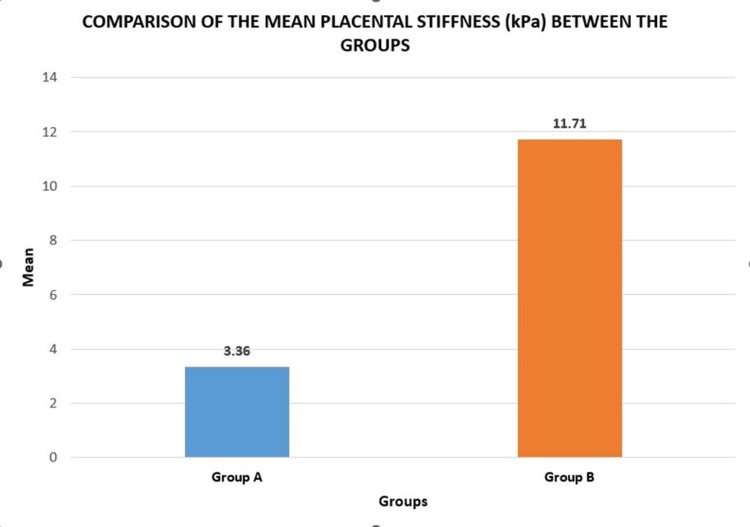
Bar graph showing comparison of the mean placental stiffness (kPa) between the groups. Group A: controls; Group B: cases

Figure 2 showing the same data in the form of a bar graph. 

## Discussion

Our study describes the role of shear wave elastography of the placenta in preeclampsia disease. The primary objective is to ascertain if there is a significant statistical difference between the tissue stiffness of the placenta in preeclamptic patients and the placenta in healthy pregnancies.

Chen et al. [6] studied healthy placentas during third trimesters using shear wave elastography. Their participants included patients between 28 and 37 weeks gestation and included 43 participants. They found no significant difference in placental stiffness as per age or period of gestation. They concluded that placental elasticity is stable in healthy placentas.

As this is a relatively newer modality, few existing studies exist. Some studies evaluated placental stiffness in healthy placentas, whereas others have studied placental stiffness in preeclampsia. Few have tried to assess patients at risk for developing preeclampsia and have followed them up to check for the development of preeclampsia.

Jingyuan Hu et al. [7] conducted a meta-analysis to review the role of shear wave elastography in the evaluation of the placenta. Their meta-analysis included 26 studies. Their study concluded that shear wave elastography of the placenta had great potential for clinical application and research involving placental function due to the objectivity and repeatable quantitative operation of shear wave elastography, especially in preeclampsia and intrauterine growth restriction (IUGR).

From our study, we concluded that the placental stiffness was significantly increased in patients with preeclampsia. Our study included 60 participants (30 diseased and 30 healthy controls). The mean age of the healthy controls was 26.13, and in the diseased group, it was 27.03. The mean difference in age between the two groups was -0.90 years, indicating that Group A was, on average, younger than Group B. However, this difference was not statistically significant, with a p-value of 0.331.

The healthy controls had gestational ages ranging from 24 to 37 weeks with a mean of 30.23 weeks. The preeclamptic group had gestational ages ranging from 26 to 37 weeks, with a mean of 31.97. The mean difference in gestational age between the two groups was -1.73 weeks, indicating that Group A had a lower average gestational age compared to Group B. However, this difference was not statistically significant, with a p-value of 0.068.

Shear wave elastography was performed on both groups, and placental stiffness was quantified. Mean placental stiffness in both groups was compared and evaluated using an independent sample t-test. The healthy control group had placental stiffness values ranging from 2.4 to 4.6 kPa, with a mean stiffness of 3.36 kPa and a standard deviation of 0.66 kPa. In contrast, the preeclamptic group had placental stiffness ranging from 8.9 to 14.8 kPa with a mean stiffness of 11.71 kPa and a standard deviation of 1.52 kPa. The mean difference in placental stiffness between the two groups was -8.34 kPa, indicating that placental stiffness increased in preeclamptic patients. This difference was statistically significant, with a p-value of 0.001.

Kılıç F. et al. [8] studied the placenta in preeclampsia and attempted to quantify the placental tissue stiffness. Their study included 50 participants (23 preeclamptic patients and 27 healthy pregnancies). The mean age in their study was 33.5 years in the preeclampsia group and 33.3 years in healthy controls. The number of participants in this study was similar to our study. The mean ages of both the preeclampsia group and healthy controls were slightly lower in our study in comparison to their study. Their study also had similar exclusion criteria as that of our own. Their study found a median value of 21 kPa for the preeclampsia group and 4 kPa for the healthy controls group. This was roughly corresponding to our own study in terms of tissue stiffness. The difference in value could be due to different equipment used and different software that each manufacturer uses. Over time, it is expected that these would be standardized.

Yan et al. [9] conducted a study in patients with hypertensive disorders of pregnancy using shear wave elastography in late-term pregnancy. They studied late-term placentas. They had 30 participants in the normal group and 22 participants in the diseased group. This number is similar to our study. They calculated the Young’s modulus, similar to our own study. They reported higher values of Young’s modulus in the placentas of diseased patients, which was in concurrence with our study.

Meena et al. [10] conducted a prospective study to evaluate if placental elastography in the second trimester can be used to predict the occurrence of preeclampsia in later gestational stages. They measured placental tissue stiffness between 16 and 20 weeks of gestation and followed up with the patients to look for the development of preeclampsia. They found that the placental stiffness was higher in the women who subsequently developed preeclampsia.

Singh et al. [11] conducted a similar study as Meena et al., wherein they studied 90 pregnancies with high-risk factors for the development of preeclampsia. They quantified placental stiffness using shear wave elastography at 20 to 24 weeks’ gestation and at 34 to 36 weeks’ gestation. The patients were then followed up to check for the development of preeclampsia. They reported that placental tissue stiffness was significantly higher in the group that went on to develop preeclampsia subsequently. 

Although these were prospective studies, the end result is in concurrence with our study, which is to say that placental tissue stiffness is higher in patients with preeclampsia.

Preeclampsia is a commonly encountered complication that has high associated morbidity and mortality, both for the mother and the fetus. It continues to be a complication that imposes a huge burden on the patient, the healthcare system, and society in terms of mortality morbidity, and healthcare costs. This implies that there is a need for a feasible and reliable tool for the prediction of preeclampsia so that proper monitoring and treatment can be given to the patient. Even after significant research, there is still significant inadequacy in the available tools with respect to early prediction and diagnosis of preeclampsia. It is based on this premise that we have studied the utility of shear wave elastography in the evaluation of preeclampsia disease.

Limitations of our study include restricted sample size, study conducted in a single hospital (could lead to bias due to single geographical location), scans done by a single radiologist (lack of inter-observer variability), shear wave elastography was conducted exclusively on patients with preeclampsia, excluding other fetal anomalies and maternal diseases that could potentially affect placental pathology, and anterior placentas can only be reliably subjected to placental shear wave elastography; hence, a significant population of preeclamptic patients with other placental locations were excluded.

## Conclusions

Hypertensive disorders of pregnancy and its dreaded complication, preeclampsia, have been significant causes of maternal/fetal mortality and morbidity. Early diagnosis remains key to managing preeclampsia so that adequate monitoring and treatment can be provided to the patients. Existing tools for the prediction of preeclampsia remain inadequate in their sensitivity and specificity. Hence, there is an urgent need for a reliable, economically feasible, and objective marker for its diagnosis/early prediction. Shear wave elastography has shown great promise in this regard. Shear wave elastography is a novel method to quantify tissue stiffness, which is objective and has significantly lower inter-observer variability. It has already been employed with great success in the evaluation of liver fibrosis. Our study showed that there is a significant statistical difference in the placental stiffness in patients with preeclampsia in comparison to a healthy placenta. This study aims to establish the significant increase in placental stiffness in patients with preeclampsia.
